# Spectral tuning of biotemplated ZnO photonic nanoarchitectures for photocatalytic applications

**DOI:** 10.1098/rsos.220090

**Published:** 2022-07-13

**Authors:** Gábor Piszter, Krisztián Kertész, Gergely Nagy, Zsófia Baji, Zsolt Endre Horváth, Zsolt Bálint, József Sándor Pap, László Péter Biró

**Affiliations:** ^1^ Institute of Technical Physics and Materials Science, Centre for Energy Research, 29-33 Konkoly Thege M. St., 1121 Budapest, Hungary; ^2^ Institute for Energy Security and Environmental Safety, Surface Chemistry and Catalysis Department, Centre for Energy Research, 29-33 Konkoly Thege M. St., 1121 Budapest, Hungary; ^3^ Department of Zoology, Hungarian Natural History Museum, 13 Baross St., 1088 Budapest, Hungary

**Keywords:** butterfly wing, photonic nanoarchitecture, structural colour, biotemplating, atomic layer deposition, photocatalysis

## Abstract

The photocatalytic activity of a flat surface can be increased by micro- and nanostructuring the interface to increase the area of the contact surface between the photocatalyst and the solute, and moreover, to optimize charge carrier transfer. Further enhancement can be achieved by using photonic nanostructures, which exhibit photonic band gap (PBG). Structurally coloured butterfly wings offer a rich ‘library’ of PBGs in the visible spectral range which can be used as naturally tuned sample sets for biotemplating. We used conformal atomic layer deposition of ZnO on the wings of various butterfly species (*Arhopala asopia*, *Hypochrysops polycletus*, *Morpho sulkowskyi*, *Polyommatus icarus*) possessing structural colour extending from the near UV to the blue wavelength range, to test the effects arising from the nanostructured surfaces and from the presence of different types of PBGs. Aqueous solutions of rhodamine B were used to test the enhancement of photocatalytic activity that was found for all ZnO-coated butterfly wings. The best reaction rate of decomposing rhodamine B when illuminated with visible light was found in 15 nm ZnO coated *M. sulkowskyi* wing, the reflectance of which had the highest overlap with the absorption band of the dye and had the highest reflectance intensity.

## 1. Introduction

Solar light is widely considered to be the primary renewable energy source for the future. Direct utilization of this renewable energy source is embodied in photocatalysis, which is an advanced oxidation/reduction process. Photocatalysis, along with advanced oxidation processes, has received significant attention due to its modest energy requirements and easy operation to break down the organic contaminants from industrial wastewater sources (dyes, pharmaceutical compounds, plastic components, etc.) into less harmful products [1]. Due to the abundance of solar light, semiconductor-based heterogeneous photocatalysis is considered one of the most encouraging technologies for resolving environmental contamination [2]. In a semiconductor-based photocatalytic process, photogenerated electrons and positive holes drive reduction and oxidation, respectively, of compounds adsorbed on the surface of a photocatalyst [3]. Successful photocatalytic reduction of drug traces on immobilized titania surfaces in municipal wastewaters has been demonstrated [4]. Recently the degradation of microplastic residues—which have nowadays become a major environmental issue due to their ubiquitous distribution, uncontrolled environmental occurrences, small sizes and long lifetimes—by a ZnO photocatalyst was reported [5].

To increase the efficiency of conversion of solar to chemical energy, it is advantageous to increase the effective surface of the photocatalyst on which the compounds can be adsorbed, and to enhance the effectivity of photoexcitation. The first goal can be achieved by replacing a flat photocatalytic surface with a micro- and possibly nanostructured surface, which on the molecular scale possesses a significantly increased effective surface. Due to their many advantages, it is foreseen that materials science will play a key role in the further development of emerging solutions for the increasing problems of energy and environment and biotemplated materials [6].

While numerous methods have been developed to produce hydro- [7] and aerogels [8] and other types of porous solids, these methods often use complex procedures and harmful substances. On the other hand, biological evolution produced materials which have complex architecture from the millimeter to the nanometer scale [9], such as the wings of butterflies covered by layers of chitinous scales [10]. Additionally, numerous butterfly species possess structural colour, i.e. coloration that arises from photonic nanoarchitectures interacting with the light falling on the wings of these butterflies [11]. These photonic nanoarchitectures act as photonic band gap (PBG) materials, which do not allow the propagation of light through them in certain wavelength ranges [11]. Therefore, in these wavelength ranges they reflect light and may produce the enhancement of photocatalytic efficiency by the so-called slow light effect [12]. Such surfaces could be useful under both aspects of enhancing photocatalytic efficiency: by increasing the effective adsorption surface and enhancing the interaction of light/photocatalyst/adsorbate to be decomposed [13–17]. Last but not least, these photonic nanoarchitectures are produced by biologic routes at ambient conditions, without the need of harmful substances and energy intensive procedures, and can be directly used in the experiments as PBG material prototypes. Many butterfly species can be reared under controlled conditions either in open air, or in artificial environments. For example, the blue Neotropical *Morpho* butterflies, which are seen in almost all butterfly-houses, or *Polyommatus icarus* [18] with Palearctic occurrence.

ZnO is one of the semiconductors which can be grown by atomic layer deposition (ALD) conformally on the scale-covered butterfly wings in such a way that the nanoscale features are preserved and can be used for photocatalysis [19,20]. Similar results can be achieved by depositing TiO_2_ coating onto butterfly wings, however, due to its large band gap, it has to be doped with metallic or non-metallic elements to expand its absorption spectrum from the UV to the visible [21–23]. The oblique-angle deposition technique, which is based on traditional vapour-deposition processes, is also a versatile tool that allows the growth of thin films comprising one-, two- or three-dimensional biological nanoarchitectures [24,25].

In the present paper, we report the use of wings of male butterflies (*Morpho sulkowskyi*, *Polyommatus icarus*, *Hypochrysops polycletus*, *Arhopala asopia*) coloured by photonic nanoarchitectures exhibiting PBG in different spectral ranges to test the photocatalytic efficiency of the biotemplated, conformal ZnO nanoarchitectures on the photocatalytic decomposition of aqueous solutions of rhodamine B (Rh B). This fluorescent compound has well-established photodegradation pathways on a variety of photoactive surfaces [26–28] and it is a representative member of the broadly used triarylmethane dyes with a xanthene core and itself is used as tracer dye in inks or in biological staining. We investigate whether differently built photonic nanoarchitectures, which for example had characteristically different vapour sensing properties [29], have different contributions to the photocatalytic activity through their geometrical differences. Therefore, we have used two types of photonic nanoarchitectures in two different colours each: multilayer-type structures (*A. asopia*, *M. sulkowskyi* [30]) and nanoporous structures (*H. polycletus* [31], *P. icarus* [10,32]), both with and without the UV reflectance component, respectively. We have found that these ‘naturally tuned’ photonic nanoarchitectures are very convenient to test the photocatalytic activity of biotemplated ZnO and to show when the reflectance peak of the structural colour has high overlap with the absorption band of the Rh B dye, the efficiency of decomposition becomes higher.

Using different biological photonic nanoarchitectures as templates, the photocatalytic properties of the deposited semiconductor layers can be efficiently explored and enhanced. Photonic nanoarchitectures of butterfly wings are excellent candidates for biotemplating as they are cheap and ready-made nanostructures produced at a macroscopic size in high quality from environmentally friendly materials. Although their mass production does not seem trivial, they are still suitable as prototypes for the experiments where artificial materials are currently not available in macroscopic sizes. The information obtained through these can be used to design bioinspired photonic nanoarchitectures that are compatible with the requirements of environmentally friendly mass production, for example, cellulose-based nanoarchitectures [33,34] which are available for roll-to-roll production preserving the convenient optical properties of the original biological templates while the semiconductor coating stabilizes their structure, making them hydrophilic and enabling efficient photocatalysis.

## 2. Results

**Figure 1.**
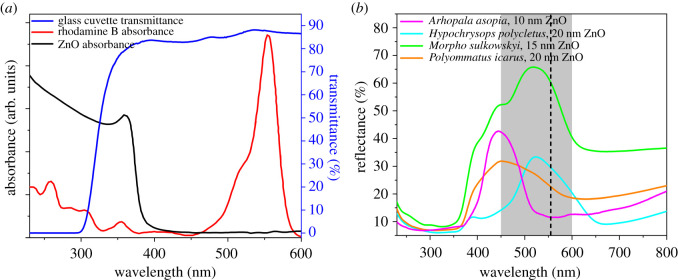
(*a*) Absorbance of ZnO [35] and Rh B [36], and the transmittance of the glass cuvette used for photodegradation measurements; (*b*) reflectance of butterfly wings conformally covered by ZnO thin films. The gray band marks the Rh B absorption range, while the dashed line indicates absorption maximum.

**Figure 2.**
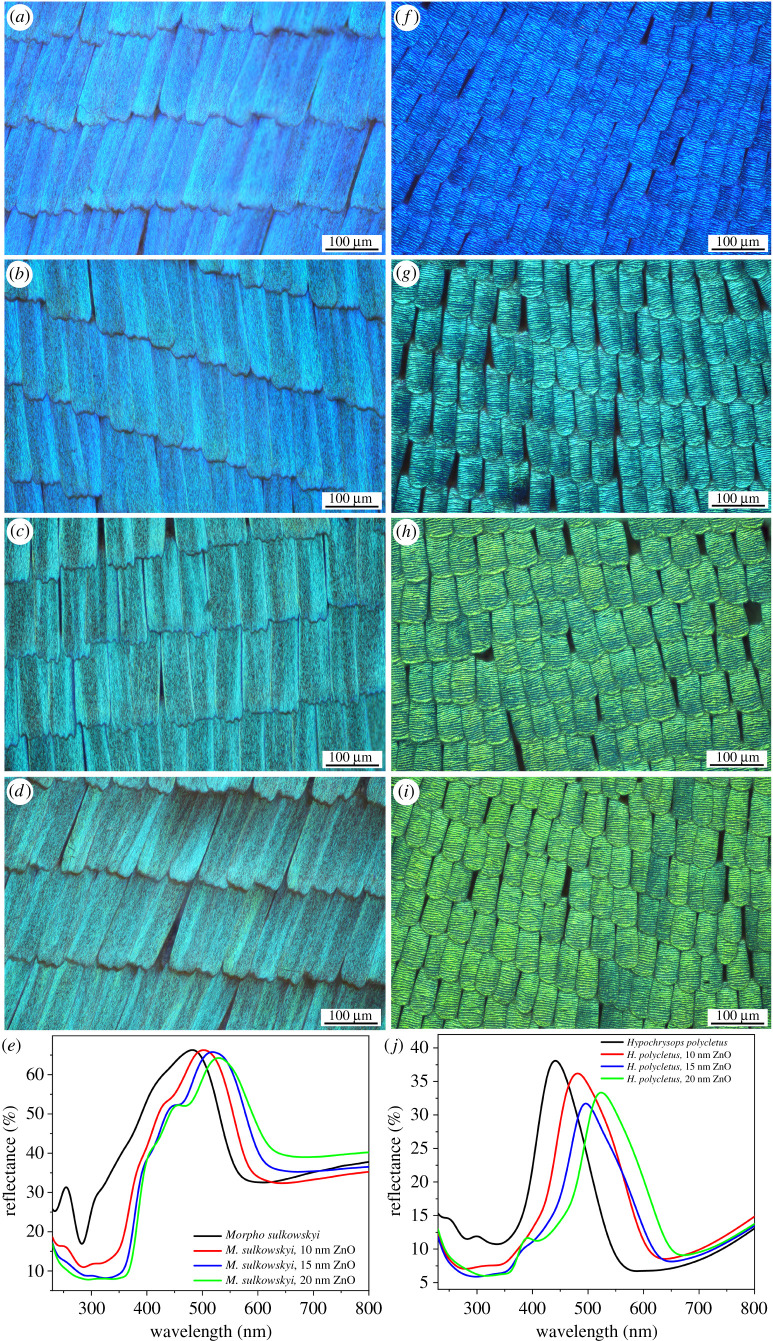
Optical microscopy and spectral characterization of (*a–e*) *Morpho sulkowskyi* and (*f–j*) *Hypochrysops polycletus* wings in pristine state and after the conformal deposition of ZnO by ALD. (*b*), (*g*) 10 nm, (*c*), (*h*) 15 nm and (*d*), (*i*) 20 nm of ZnO layer thicknesses are shown. The corresponding reflectance spectra are shown in (*e*) and (*j*), respectively.

In figure 1*a*, the absorbance of ZnO thin film [35] and Rh B [36] as known from the literature, and the transmittance of the glass cuvette, in which the experiments were carried out, are plotted. The transmittance of the glass cuvette reaches 80% at 360 nm and increases to 85% at 600 nm. The spectrum of the light source is shown in electronic supplementary material, figure S1. The reflectance of four butterfly wings: *Arhopala asopia*, *Hypochrysops polycletus*, *Morpho sulkowskyi* and *Polyommatus icarus* conformally coated by ALD with variable thicknesses of ZnO are plotted in figure 1*b*. These ALD coated butterfly wings, as seen in figure 1*b*, have different maxima of the reflectance peak and also exhibit different overlaps with the absorption peak of Rh B. One may note that when the ZnO layer was applied, the spectral features of the butterfly wings were completely masked by light absorption of the ZnO layer with an absorption edge of 380 nm (see also figure 2*e,j*). The electronic band gap of the deposited ZnO layers was estimated to be 3.3–3.35 eV by transmittance measurements (electronic supplementary material, figure S2).

Due to the low temperature reaction, the conformal deposition of ZnO by ALD did not adversely affect the morphology and the regular arrangement—like tiles on a roof—of the cover scales on the wing surfaces of the butterflies. See, for example, the optical microscopy images in figure 2*a–d* for the *M. sulkowskyi* and figure 2*f–i* for the *H. polycletus* wings. In agreement with the data reported earlier [37], the maximum of the reflectance peak was redshifted with the thickening of the deposited ZnO layer (figure 2*e,j*). As ZnO thin films have a refractive index of 1.5–1.6 in the visible wavelength range [38], the deposition resulted in the increased thickness of the high refractive index (chitinous) component, meanwhile decreasing the thickness of the low refractive index component (air voids) of the photonic nanoarchitecture, causing the redshift of the reflectance.

**Figure 3.**
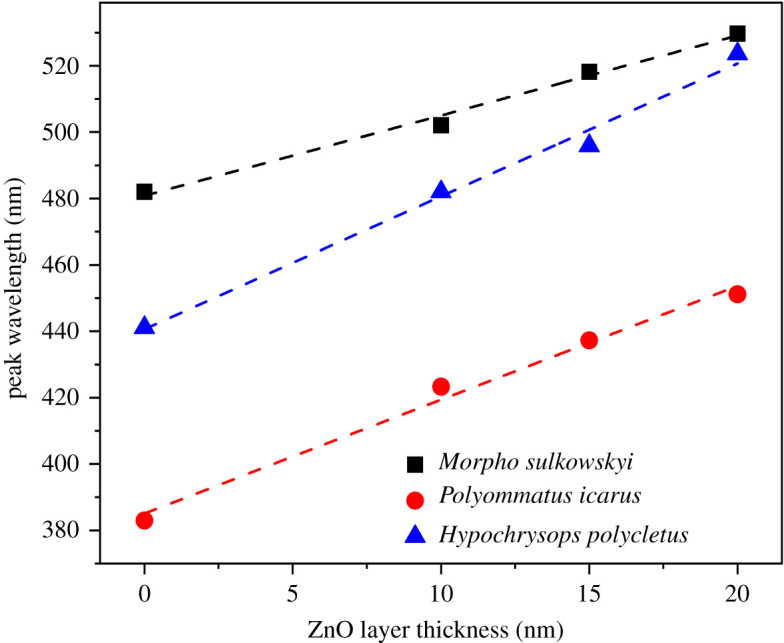
Peak shift of the reflectance of different butterfly wings with the increasing thickness of the deposited ZnO layer. *Hypochrysops polycletus*, *Morpho sulkowskyi* and *Polyommatus icarus* are shown in pristine states and when 10–15–20 nm of ZnO coatings were deposited.

The two types of photonic nanoarchitectures exhibited different magnitude shifts with the increasing thickness of the ZnO layer (compare the two columns in figure 2). The nanoporous structure of *H. polycletus* although, also a regular photonic nanoarchitecture, is very different from the multilayer-type structure of the *M. sulkowskyi* [30]. When comparing the behaviour of *M. sulkowskyi* [30], *H. polycletus* [31] and *P. icarus* scales [10,32], the latter two show similarity in the spectral shift. In fact, their nanoarchitectures, although not identical, exhibit similar nanoporous structure, while both are very different from the *Morpho*–type multilayer scales. The different redshifts for the three different species are summarized in figure 3. For *P. icarus* and *H. polycletus* the plateau over which the spectral position of the reflectance maximum could be positioned is of 80 nm, while for *M. sulkowskyi*, it is of the order of 50 nm.

**Figure 4.**
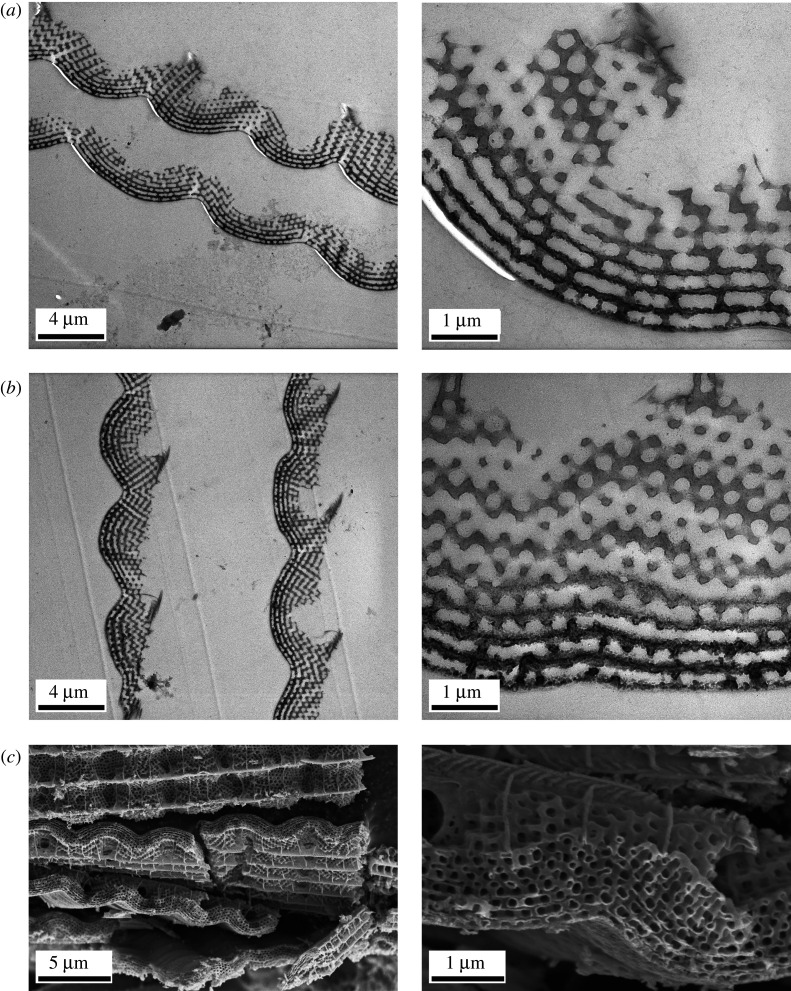
Cross-sectional TEM and SEM images of the cover scales of *Hypochrysops polycletus* (*a*) before and (*b*) after 20 nm ZnO deposition. One may observe that neither the micrometer scale, nor the nanometer-scale structure was affected. (*c*) The conformal ZnO coverage of 20 nm is shown after the chitinous template was removed by oxidation in air for 3 h at 500°C.

The cross-sectional TEM images of pristine and ALD coated *H. polycletus* scales and the conformal coating by ALD, as revealed after the oxidative removal of the chitinous material of the scale, are shown in figure 4. One may observe that as reported in [6,20] for *M. sulkowskyi*, and in [37] for *P. icarus*, the deposition process does not damage the pristine photonic nanoarchitecture of *H. polycletus,* the coating is continuous and self-supporting to the degree which allows the complete removal of the original chitinous nanoarchitecture by oxidation.

**Figure 5.**
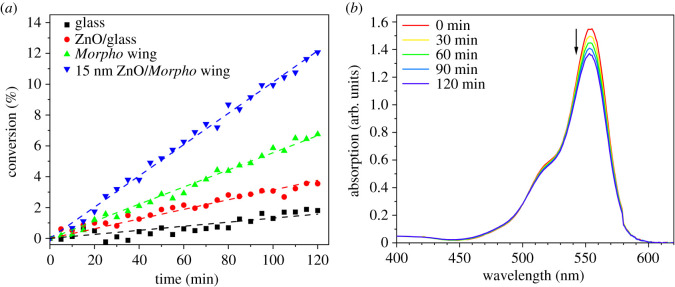
(*a*) Reaction rate versus time for bare glass, ZnO covered glass, pristine *Morpho sulkowskyi* wing and 15 nm ZnO covered *M. sulkowskyi* wing. (*b*) Absorption spectra of Rh B measured during the 120-minute-long photodecomposition measurement on the 15 nm ZnO-covered *M. sulkowskyi* wing.

To demonstrate the combined effect of the photonic nanoarchitecture and the photocatalytic coating by ZnO on the conversion rate of the photodecomposition of Rh B over uncoated glass, ZnO coated glass, pristine *M. sulkowskyi* wing and *M. sulkowskyi* wing with 15 nm conformal ZnO coating was compared as a function of reaction time (figure 5). The ZnO coating increased the reaction rate in the case of flat glass by a factor of 2.3, and for ZnO coated *M. sulkowskyi* wing as compared with the pristine wing by a factor of almost 2. It is worth pointing out that the ZnO coated wing exhibited a roughly three-fold increase as compared to the same ZnO layer on glass substrate.

## 3. Discussion

The photocatalytic activity of butterfly wings coloured by photonic nanoarchitectures that exhibit PBG in the visible were investigated both in pristine state and after conformal coating with ALD grown ZnO. The status and reaction rates of the samples discussed are summarized in table 1. Note that these reaction rates were calculated uniformly for the first two hours of reaction time, in which period the conversion of Rh B was low enough to enable the conversion versus time data points be fitted with a linear (figure 5). A uniform and homogeneous light intensity was allowed by the lamp and reactor setup and due to the planar geometry of the butterfly wings (and glass pieces), the photocatalytic activity could be directly compared based on the geometric area rather than normalization by mass, which is typically used for powdery catalysts. Air-saturated aqueous solutions of Rh B dye in a glass cuvette were used as test reactor. The possibility of using the visible spectral range for photodecomposition is important if large-scale practical applications are envisaged (as sunlight maximum is in the visible), the necessity of quartz reactors instead of glass [39] could significantly increase the price of photocatalytic installations.

**Table 1.** Status and reaction rates of the glass and butterfly-based samples discussed in the text in detail.

sample	plasma treatment	ZnO layer thickness (nm)	reaction rate (nmol min^−1^)
glass	no	0	0.039(3)
glass	no	20	0.093(2)
*Morpho sulkowskyi*	no	0	0.167(2)
*Morpho sulkowskyi*	no	15	0.303(6)
*Morpho sulkowskyi*	3 min	20	0.150(3)
*Hypochrysops polycletus*	no	0	0.162(3)
*Hypochrysops polycletus*	no	20	0.210(1)
*Polyommatus icarus*	no	0	0.087(2)
*Polyommatus icarus*	no	10	0.165(5)
*Polyommatus icarus*	no	20	0.189(2)
*Arhopala asopia*	no	10	0.210(1)

ZnO is a very attractive substance as it can be conveniently deposited conformally by a low temperature ALD process on biological templates. The comparison of reaction rates (figure 5) for unstructured glass, and butterfly wings containing photonic nanoarchitectures, clearly shows that by biotemplating nanoarchitectures and exploiting the effect of the PBG, significant increase of the reaction rate can be achieved, up to 3.3 times if compared to ZnO coated glass, despite the fact that the absorption band of Rh B falls outside of the spectral region where ZnO absorbs light (figure 1*a*). This increase is associated with slow light effects [17] arising due to the presence of the photonic nanoarchitecture of biologic origin, as the red edge of the PBG is active from a slow light perspective [40]. As seen in figure 1*b*, the absorption band of Rh B and the reflectance of the *Morpho sulkowskyi* wing covered by 15 nm conformal ZnO show a good overlap, while it also preserves its intensive structural colour. These two properties together result in the best photocatalytic efficiency in this experiment. Therefore, the different *Morpho* species [41], possessing intensive structural colours of different blues, may offer a variety of biophotonic structures to be used for the spectral tuning of the enhancement effect in the desired spectral range, and also these *Morpho* wings can be effectively used as templates for controlled replication for photocatalysis [42].

Another method of spectral tuning is to use different layer thicknesses during the deposition of ZnO by ALD. As seen in figures 2 and 3, the position of the reflectance maxima can be tuned over a wavelength range of 50 to 80 nm only by selecting the desired layer thickness. A further tuning possibility arises from the very rich ‘library’ of butterfly species possessing structural colour. For example, the males of many Gossamer-winged butterflies (Lepidoptera: Papilionoidea: Lycaenidae)—one of the most speciose butterfly families with 416 genera and 5201 species [43]—possess species-specific blue sexual signalling colours of structural origin. In a study on *Polyommatus* Latreille, 1804 subgenus *Agrodiaetus* Hübner, 1822 from 140 species in the tribe *Polyommatini* (*Polyommatinae*), only 26 species were found with brown dorsal coloration of the males, with all the others exhibiting different structural colours [44]. These colours, as they have a role in sexual selection of mates [45], are reproduced with very high precision over time periods of 100 years and distances of many thousands of kilometers [46,47]. And last, but not least, a further tuning possibility is offered by the combination of materials science methods like plasma etching of chitin and deposition of ZnO by ALD on the etched photonic nanoarchitecture [37].

The combination of plasma etching (see details in [37]) with the deposition of conformal ZnO by ALD may equally well improve or deteriorate the photocatalytic efficiency of the biotemplated photocatalyst. For example, the *M. sulkowskyi* wing treated by oxygen plasma etching for 3 min followed by a 20 nm conformal ALD layer of ZnO has half the photocatalytic efficiency, 0.150 nmol min^−1^, as compared with the pristine wing covered by 15 nm of conformal ZnO, 0.303 nmol min^−1^. In fact, this value is closer to that of the unstructured glass covered by ZnO. When comparing the reflectance of the two differently processed *Morpho* wings (electronic supplementary material, figure S3), one may observe that the plasma treatment followed by ALD almost completely eliminated the PBG of the original photonic nanoarchitecture. This underscores the important role of the photonic nanoarchitecture in the enhancement of photocatalytic efficiency.

**Figure 6.**
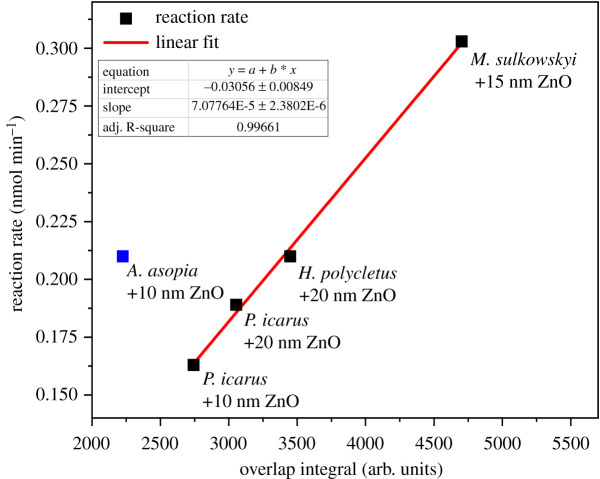
Reaction rate as a function of the overlap between the wing reflectance spectra of the biotemplated samples and the absorption spectrum of Rh B (figure 1*b*). Linear relationship was found between the overlap integral and the photocatalytic activity except for *Arhopala asopia*, where higher-than-expected reaction rate was measured, and thus it was left out from the fit.

In the case of the *Hypochrysops polycletus* wings, the conformal ZnO coating of 20 nm redshifts the reflectance maximum in such a way that it will have a much better overlap with the absorption band of the Rh B (figure 2). This yields an increase in the decomposition rate of the dye from 0.162 nmol min^−1^ to 0.210 nmol min^−1^, clearly showing that apart from the spectral overlap (figure 1*b*), the high-intensity reflectance is also important for the efficient photocatalysis (figure 6).

For the pristine *P. icarus* wings only a decomposition rate of 0.087 nmol min^−1^ was found, which is comparable to the value for unstructured glass covered by 20 nm ZnO, 0.093 nmol min^−1^. The reflectance of uncoated *P. icarus* wings has the maximum close to 33% around 390 nm and drops to about 15% at 550 nm [48]. The deposition of 10 nm of conformal ZnO increases the reaction rate to twice the value for uncoated wing, 0.165 nmol min^−1^. The deposition of 20 nm of ZnO increases the decomposition rate to 0.189 nmol min^−1^. This increase is attributed to the redshift of the reflectance maximum so that a much better overlap is achieved with the absorption of Rh B, figure 1*b*, but still, the relatively low reflectance maximum limits the photocatalytic efficiency (figure 6).

In figure 1*b*, the spectra of four coated wings are compared: *M. sulkowskyi* with 15 nm ZnO, *A. asopia* with 10 nm ZnO, *H. polycletus* with 20 nm ZnO, and *P. icarus* with 20 nm ZnO. The corresponding decomposition rates are: 0.303 nmol min^−1^, 0.210 nmol min^−1^, 0.210 nmol min^−1^ and 0.189 nmol min^−1^, respectively. The reaction rate is proportional to the intensity and to the overlap between the red edge of the wing reflectance and the absorption of the dye (figure 6), except for *A. asopia*, for which higher-than-expected photocatalytic activity was observed.

Under photocatalytic conditions, the photogenerated holes can react with water, forming hydroxyl radicals as strong oxidant, while the dissolved O_2_ can capture the conduction band (CB) electrons to form the reactive superoxide radical anion. In addition to the holes, these radicals are also able to attack the dye molecules causing degradation. This route of photocatalytic degradation can be elucidated by using hydroquinone (H_2_Q) [49–53] that does not absorb light in the reflectance band region of the 15 nm ZnO-coated *M. sulkowskyi* wing.

The photolytic autooxidation of H_2_Q in the presence of O_2_ is expected to set an equilibrium between H_2_Q and benzoquinone (BQ), also producing some hydroxylated products like 2,5-dihydroxy-1,4-benzoquinone (2,5-HO-BQ) in the pH range of 4–7. Thus, the sum of the concentrations of BQ, H_2_Q and the hydroxylated derivatives remains constant, and no ring-cleavage and mineralization occur. In this case, the UV-visible absorption spectrum exhibits bands at *λ*_max_ of 288 nm (H_2_Q), 254 nm (BQ) and approximately 490 nm (2,5-HO-BQ). Note that quinhydrone may also form and absorb in the visible region.

**Figure 7.**
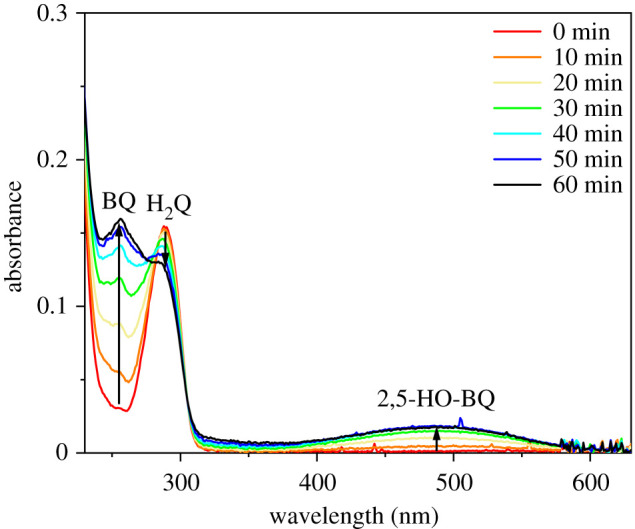
UV-visible absorption spectra recorded in the course of hydroquinone (H_2_Q, 60 µM) photolysis in the presence of 15 nm ZnO-coated *Morpho sulkowskyi* wing. Conditions are identical to the photocatalytic conditions applied for Rh B.

On the other hand, under oxic photocatalytic conditions, the benzene ring of BQ undergoes direct hydroxylation and further transformation to semiquinone radicals that finally results in various hydroxylated intermediates and rapid mineralization [49]. This process would be unequivocally indicated by a complete loss in the absorption bands that is clearly not the case with the ZnO-coated *M. sulkowskyi* wing (figure 7), thus excluding the role of photogenerated oxygen radicals in the degradation of dye under our reaction conditions. By contrast, the fact that the colourless H_2_Q does not undergo rapid mineralization makes the contribution of dye sensitization by Rh B to the photocatalytic degradation [40] a more likely scenario in our case.

More experiments are needed to explore the relationship between the structural colours and photocatalytic activity in detail by testing further dyes with tuned biotemplated photonic nanoarchitectures.

## 4. Material and methods

### 4.1. Butterflies

The butterfly samples were obtained from the collection of the Institute of Technical Physics and Materials Science, Centre for Energy Research. Male specimens of *Arhopala asopia* (Lycaenidae: Arhopalini), *Hypochrysops polycletus* (Lycaenidae: Luciini) [31], *Morpho sulkowskyi* (Nymphalidae: Morphini) [30], *Polyommatus icarus* (Lycaenidae: Polyommatini) [10,32] were investigated. None of the species used in this study were subjected to any restrictions.

### 4.2. Atomic layer deposition (ALD)

Atomic layer deposition of 10, 15 and 20 nm thick ZnO layers was carried out in a Picosun Sunale R-100 ALD reactor. Diethylzinc (DEZ) precursor and water vapour as oxidant were used for the deposition. The carrier gas and purging medium was 99.999% purity nitrogen. Flow rates of the precursor gas and water were 150 sccm. During deposition, the pressure in the chamber was kept at 14 mbar. An ALD cycle for depositing ZnO layers consisted of a 0.5 s pulse of DEZ and 15 s nitrogen purge, followed by a 0.5 s pulse of water and 20 s nitrogen purge. As the wing samples were thermally sensitive, the growth temperature was maintained at 100°C. The growth rate at this temperature was 0.18 nm cycle^−1^, therefore the 10 nm layer thickness required 60 pulses, the 15 nm layer 90 pulses and the 20 nm layer 120 pulses.

The glass substrates (used as flat, reference surface) were prepared by cutting them into the desired size and were cleaned with acetone, isopropyl alcohol, and deionized water. The same ZnO coating process was applied as in the case of the butterfly wings.

For the removal of chitinous nanoarchitecture from the ZnO coated samples, the wing pieces were thermally treated for 3 h at 500°C in a furnace using air atmosphere.

### 4.3. Microscopy

Optical microscope images were taken with the ×20 objective of a Nikon Eclipse LV150N (Shinagawa, Tokyo, Japan) device using extended depth of focus (EDF) mode which resulted in high depth of field images of the otherwise significantly textured butterfly wing surfaces.

The butterfly wing samples were prepared for electron microscopy using standard techniques [10]. The samples were examined via scanning electron microscopy (SEM) and cross-sectional transmission electron microscopy (TEM) imaging using Thermo Fisher Scientific Scios 2 DualBeam (Waltham, MA, USA) and Philips CM20 (Eindhoven, The Netherlands) systems, respectively.

### 4.4. Reflectance spectroscopy

Optical reflectance measurements were carried out using a fibre optic Avantes (Apeldoorn, The Netherlands) system consisting of an AvaSpec-HERO spectrophotometer, an AvaLight-DH-S-BAL stabilized UV-visible light source, an integrating sphere (AvaSphere-30-REFL) for light collection, and a WS-2 diffuse tile as a reference. Reflectance of pristine and ZnO coated wing pieces were measured in 200–950 nm wavelength range. Data analysis was performed using OriginPro 2021 (OriginLab Corporation, Northampton, MA, USA) software.

### 4.5. Photocatalytic degradation of rhodamine B (Rh B)

The photocatalytic activity was evaluated based on the removal of Rh B (15 µM) from its unbuffered solution upon illumination using MilliQ ultrapure water as solvent. The ZnO samples were placed vertically in 20 ml of the Rh B solution in a glass cuvette with magnetic stirring at room temperature. A heat free 300 W xenon lamp (Asahi Spectra MAX-301, Torrance, CA, USA) with fibre optics was applied as a light source supplying an adjustable square-shaped beam (1.5 × 1.5 cm square-shaped illumination). The distance between the lamp and the catalytic surface immersed in the cuvette was 6 cm (corresponding to approx. 100 mW cm^−2^ light power). The rate of degradation was followed by an Agilent Cary 60 (Santa Clara, CA, USA) UV–visible spectrophotometer equipped with an immersion probe (*l* = 1 cm) that was placed inside the solution out of the illumination area. Spectra were collected every fifth minute during the 2 h of reaction time. Degradation of Rh B was followed at the wavelength of its absorption maximum at 554 nm. Conversion was calculated as (A_0_-A_i_)/A_0_, where A_0_ is the initial absorbance of Rh B at 554 nm, and A_i_ is the absorbance at a given point in the reaction. Soaking tests of the surfaces in Rh B solution before their use ruled out any detectable role of initial adsorption of the dye in the change of absorbance (note the high reaction volume over catalytic surface area ratio).

## 5. Conclusion

All the tested butterfly wings coloured by photonic nanoarchitectures in pristine state exhibited some increase as compared with the decomposition rate corresponding to a flat glass surface. This may be attributed to the increased effective surface and to the more intense light field at the surface of the nanoarchitecture exhibiting PBG. All the samples exhibited an increase of the decomposition rate after the deposition of a conformal ZnO layer by ALD. This deposition process fully conserved the micro- and the nanoscale structure of the butterfly wings. After the deposition of the ZnO, all samples exhibited increased reaction rate compared to the pristine samples. The magnitude of this increase depends on several factors: (i) the overlap between the red edge of reflectance peak of the photonic nanoarchitecture resulted from the biotemplated ZnO nanolayer and the absorption band of the test dye; (ii) the magnitude of the reflectance peak of the complex photonic nanoarchitecture. The spectral position of the reflectance of the photonic nanoarchitecture can be tuned by the thickness of the deposited ZnO and by using oxygen plasma tailoring of the photonic nanoarchitecture before the ZnO deposition. The experimental results show that enhancement of the decomposition of the test dye can be produced even when most of the UV light is excluded (roughly above 360 nm). This indicates that such biotemplated ZnO photonic nanoarchitectures may offer a more efficient route to photocatalysis than systems that use quartz for transparency in the UV.

## Data Availability

All supporting data are available in the electronic supplementary material [54]. Experimental data can be accessed from the Dryad Digital Repository: https://doi.org/10.5061/dryad.w9ghx3fr8 [55].

## References

[1] Byrne C, Subramanian G, Pillai SC. 2018 Recent advances in photocatalysis for environmental applications. J. Environ. Chem. Eng. **6**, 3531-3555. (10.1016/j.jece.2017.07.080)

[2] Ansari MO, Kumar R, Ansari SP, Hassan MSA, Alshahrie A, Barakat MAE. 2019 Nanocarbon aerogel composites. In Nanocarbon and Its composites: preparation, properties and applications (eds A Khan, M Jawaid, AMA Asiri), pp. 1-26. Sawston, UK: Woodhead Publishing.

[3] Ohtani B. 2011 Photocatalysis by inorganic solid materials. In Advances in inorganic chemistry, vol. 63 (eds R van Eldik, G Stochel), pp. 395-430. Amsterdam, The Netherlands: Elsevier.

[4] Miranda-García N, Suáraz S, Sánchez B, Coronado JM, Malato S, Maldonado MI. 2011 Photocatalytic degradation of emerging contaminants in municipal wastewater treatment plant effluents using immobilized TiO_2_ in a solar pilot plant. Appl. Catal. B Environ. **103**, 294-301. (10.1016/j.apcatb.2011.01.030)

[5] Tofa TS, Kunjali KL, Paul S, Dutta J. 2019 Visible light photocatalytic degradation of microplastic residues with zinc oxide nanorods. Environ. Chem. Lett. **17**, 1341-1346. (10.1007/s10311-019-00859-z)

[6] Zhou H, Fan T, Zhang D. 2011 Biotemplated materials for sustainable energy and environment: current status and challenges. ChemSusChem **4**, 1344-1387. (10.1002/cssc.201100048) 21905237

[7] Ahmed EM. 2015 Hydrogel: preparation, characterization, and applications: a review. J. Adv. Res. **6**, 105-121. (10.1016/j.jare.2013.07.006) 25750745 PMC4348459

[8] Soleimani DA, Abbasi MH. 2008 Silica aerogel; synthesis, properties and characterization. J. Mat. Proc. Tech. **199**, 10-26. (10.1016/j.jmatprotec.2007.10.060)

[9] Sharna V, Kumar S, Reddy KL, Bahuguna A, Krishnan V. 2016 Bioinspired functional surfaces for technological applications. J. Mol. Eng. Mater. **4**, 1640006. (10.1142/S2251237316400062)

[10] Bálint Z, Kertész K, Piszter G, Vértesy Z, Biró LP. 2012 The well-tuned blues: the role of structural colours as optical signals in the species recognition of a local butterfly fauna (Lepidoptera: Lycaenidae: Polyommatinae). J. R. Soc. Interface **9**, 1745-1756. (10.1098/rsif.2011.0854) 22319114 PMC3385757

[11] Biró LP, Vigneron JP. 2011 Photonic nanoarchitectures in butterflies and beetles: valuable sources for bioinspiration. Laser Photon. Rev. **5**, 27-51. (10.1002/lpor.200900018)

[12] Liu J, Wu M, Van der Schueren B, Deparis O, Ye J, Ozin GA, Hasan T, Su BL. 2017 Slow photons for photocatalysis and photovoltaics. Adv. Mater. **29**, 1605349. (10.1002/adma.201605349) 28165167

[13] Sharma V, Balaji R, Kumar A, Kumari N, Krishnan V. 2018 Bioinspired 3D surface-enhanced raman spectroscopy substrates for surface plasmon driven photoxidation reactions: role of catalyst and substrate in controlling the selectivity of product formation. ChemCatChem **10**, 975-979. (10.1002/cctc.201701616)

[14] Sharma V, Bahuguna A, Krishnan V. 2017 Bioinspired dip catalysts for Suzuki–Miyaura cross-coupling reactions: effect of scaffold architecture on the performance of the catalyst. Adv. Mater. Interfaces **4**, 1700604. (10.1002/admi.201700604)

[15] Sharma V, Kumar S, Bahuguna A, Gambhir D, Sagara PS, Krishnan V. 2017 Plant leaves as natural green scaffolds for palladium catalyzed Suzuki–Miyaura coupling reactions. Bioinspir. Biomim. **12**, 016010. (10.1088/1748-3190/12/1/016010) 28000624

[16] Kumari N, Kumar A, Krishnan V. 2021 Ultrathin Au–Ag heterojunctions on nanoarchitectonics based biomimetic substrates for dip catalysis. J. Inorg. Organomet. Polym. Mater. **31**, 1954-1966. (10.1007/s10904-021-01902-9)

[17] Kumari N, Sood N, Krishnan V. 2022 Beetle wing inspired fabrication of nanojunction based biomimetic SERS substrates for sensitive detection of analytes. Mater. Technol. **37**, 112-123. (10.1080/10667857.2020.1816382)

[18] Kertész K, Piszter G, Horváth ZE, Bálint Z, Biró LP. 2017 Changes in structural and pigmentary colours in response to cold stress in *Polyommatus icarus* butterflies. Sci. Rep. **7**, 1118. (10.1038/s41598-017-01273-7) 28442788 PMC5430924

[19] Kumar S, Kumar A, Kumar A, Krishnan V. 2020 Nanoscale zinc oxide based heterojunctions as visible light active photocatalysts for hydrogen energy and environmental remediation*.* Catal. Rev. **62**, 346-405. (10.1080/01614940.2019.1684649)

[20] Rodríguez RE, Agarwal SP, An S, Kazyak E, Das D, Shang W, Skye R, Deng T, Dasgupta NP. 2018 Biotemplated *Morpho* butterfly wings for tunable structurally colored photocatalysts. ACS Appl. Mater. Interfaces **10**, 4614-4621. (10.1021/acsami.7b14383) 29337532

[21] Mihai S, Cursaru DL, Ghita D, Dinescu A. 2016 Morpho ierarhic TiO_2_ with plasmonic gold decoration for highly active photocatalysis properties. Mater. Lett. **165**, 222-225. (10.1016/j.matlet.2015.10.012)

[22] Chen J, Su H, Song F, Moon W-J, Kim Y-S, Zhang D. 2012 Bioinspired Au/TiO_2_ photocatalyst derived from butterfly wing (*Papilio paris*). J. Colloid Interface Sci. **370**, 117-223. (10.1016/j.jcis.2011.12.055) 22244864

[23] Wang Y, Xiong D-B, Zhang W, Su H, Liu Q, Gu J, Zhus S, Zhang D. 2016 Surface plasmon resonance of gold nanocrystals coupled with slow-photon-effect of biomorphic TiO_2_ photonic crystals for enhanced photocatalysis under visible-light. Catal. Today **274**, 15-21. (10.1016/j.cattod.2016.01.05)

[24] Plawsky JL, Kim JK, Schubert EF. 2009 Engineered nanoporous and nanostructured films. Mater. Today **12**, 36-45. (10.1016/S1369-7021(09)70179-8)

[25] Martin-Palma RJ, Lakhtakia A. 2014 Oblique-angle deposition: evolution from sculptured thin films to bioreplication. Scr. Mater. **74**, 9-12. (10.1016/j.scriptamat.2013.06.006)

[26] Wilhelm P, Stephan D. 2007 Photodegradation of rhodamine B in aqueous solution via SiO_2_@TiO_2_ nano-spheres. J. Photochem. Photobiol. A **185**, 19-25. (10.1016/j.jphotochem.2006.05.003)

[27] Wang Q, Lian J, Ma Q, Bai Y, Tong J, Zhong J, Wang R, Huang H, Su B. 2015 Photodegradation of Rhodamine B over a novel photocatalyst of feather keratin decorated CdS under visible light irradiation. New J. Chem. **39**, 7112-7119. (10.1039/C5NJ00987A)

[28] Lee SY, Kang D, Jeong S, Do HT, Kim JH. 2020 Photocatalytic degradation of Rhodamine B dye by TiO_2_ and gold nanoparticles supported on a floating porous polydimethylsiloxane sponge under ultraviolet and visible light irradiation. ACS Omega **5**, 4233-4241. (10.1021/acsomega.9b04127) 32149253 PMC7057704

[29] Piszter G, Kertész K, Bálint Z, Biró LP. 2019 Optical detection of vapor mixtures using structurally colored butterfly and moth wings. Sensors **19**, 3058. (10.3390/s19143058) 31336702 PMC6678582

[30] Prum RO, Quinn T, Torres RH. 2006 Anatomically diverse butterfly scales all produce structural colours by coherent scattering. J. Exp. Biol. **209**, 748-765. (10.1242/jeb.02051) 16449568

[31] Ingram AL, Parker AR. 2008 A review of the diversity and evolution of photonic structures in butterflies, incorporating the work of John Huxley (The Natural History Museum, London from 1961 to 1990*)*. Phil. Trans. R. Soc. B **363**, 2465-2480. (10.1098/rstb.2007.2258) 18331987 PMC2606806

[32] Piszter G, Kertész K, Horváth ZE, Bálint Z, Biró LP. 2019 Reproducible phenotype alteration due to prolonged cooling of the pupae of *Polyommatus icarus* butterflies. PLoS ONE **14**, e0225388. (10.1371/journal.pone.0225388) 31765404 PMC6876796

[33] Liang HL, Bay MM, Vadrucci R, Barty-King CH, Peng J, Baumberg JJ, De Volder MFL, Vignolini S. 2018 Roll-to-roll fabrication of touch-responsive cellulose photonic laminates. Nat. Commun. **9**, 4632. (10.1038/s41467-018-07048-6) 30401803 PMC6219516

[34] Droguet BE, Liang HL, Frka-Petesic B, Parker RM, De Volder MFL, Baumberg JJ, Vignolini S. 2022 Large-scale fabrication of structurally coloured cellulose nanocrystal films and effect pigments. Nat. Mater. **21**, 352-358. (10.1038/s41563-021-01135-8) 34764430

[35] Yu J, Shan C-X, Qiao Q, Xie X-H, Wang S-P, Zhang Z-Z, Shen D-Z. 2012 Enhanced Responsivity of Photodetectors Realized via Impact Ionization. Sensors **12**, 1280-1287. (10.3390/s120201280) 22438709 PMC3304111

[36] Bartasun P, Cieśliński H, Bujacz A, Wierzbicka-Woś A, Kur J. 2013 A Study on the interaction of Rhodamine B with methylthioadenosine phosphorylase protein sourced from an Antarctic soil metagenomic library. PLoS ONE **8**, e55697. (10.1371/journal.pone.0055697) 23383268 PMC3561333

[37] Kertész K, Baji Z, Deák A, Piszter G, Rázga Z, Bálint Z, Biró LP. 2021 Additive and subtractive modification of butterfly wing structural colors. Colloid Interface Sci. Commun. **40**, 100346. (10.1016/j.colcom.2020.100346)

[38] Iqbal J, Jilani A, Ziaul Hassan PM, Rafique S, Jafer R, Alghamdi AA. 2016 ALD grown nanostructured ZnO thin films: effect of substrate temperature on thickness and energy band gap. J. King Saud. Univ. Sci. **28**, 347-354. (10.1016/j.jksus.2016.03.001)

[39] Kim S, Cho H, Joo H, Her N, Han J, Yi K, Kim J-O, Yoon J. 2017 Evaluation of performance with small and scale-up rotating and flat reactors; photocatalytic degradation of bisphenol A, 17β–estradiol, and 17α–ethynyl estradiol under solar irradiation. J. Hazard. Mater. **336**, 21-32. (10.1016/j.jhazmat.2017.04.047) 28463735

[40] Lim SY, Hedrich C, Jiang L, Law CS, Chirumamilla M, Abell AD, Blick RH, Zierold R, Santos A. 2021 Harnessing slow light in optoelectronically engineered nanoporous photonic crystals for visible light-enhanced photocatalysis. ACS Catalysis **11**, 12 947-12 962. (10.1021/acscatal.1c03320)

[41] Berthier S. 2010 Photonique des morphos, 300pp. Paris, France: Springer.

[42] Zhu M, Zhang H, Favier SWL, Zhao Y, Guo H, Du Z. 2021 A general strategy towards controllable replication of butterfly wings for robust light photocatalysis. J. Mat. Sci. Tech. **105**, 286-292. (10.1016/j.jmst.2021.07.035)

[43] van Nieukerken EJ et al. 2011 Order Lepidoptera Linnaeus, 1758. In: Zhang, Z.-Q. (Ed.) Animal biodiversity: an outline of higher-level classification and survey of taxonomic richness. Zootaxa **3148**, 212. (10.11646/zootaxa.3148.1.41) 26146682

[44] Wiemers M, Keller A, Wolf M. 2009 ITS2 secondary structure improves phylogeny estimation in a radiation of blue butterflies of the subgenus *Agrodiaetus* (Lepidoptera: Lycaenidae: Polyommatus). BMC Evol. Biol. **9**, 300. (10.1186/1471-2148-9-300) 20035628 PMC2810301

[45] Lukhtanov VA, Kandul NP, Plotkin JB, Dantchenko AV, Haig D, Pierce NE. 2005 Reinforcement of pre-zygotic isolation and karyotype evolution in *Agrodiaetus* butterflies. Nature **436**, 385-389. (10.1038/nature03704) 16034417

[46] Kertész K, Piszter G, Bálint Z, Biró LP. 2019 Biogeographical patterns in the structural blue of male *Polyommatus icarus* butterflies. Sci. Rep. **9**, 2338. (10.1038/s41598-019-38827-w) 30787341 PMC6382816

[47] Piszter G, Kertész K, Sramkó G, Krízsik V, Bálint Z, Biró LP. 2011 Concordance of the spectral properties of dorsal wing scales with the phylogeographic structure of European male *Polyommatus icarus* butterflies. Sci. Rep. **11**, 16498. (10.1038/s41598-021-95881-z) PMC8363635 34389765

[48] Piszter G, Kertész K, Bálint Z, Biró LP. 2016 Variability of the structural coloration in two butterfly species with different prezygotic mating strategies. PLoS ONE **11**, e0165857. (10.1371/journal.pone.0165857) 27832120 PMC5104395

[49] Fónagy O, Szabó-Bárdos E, Horváth O. 2021 1,4-Benzoquinone and 1,4-hydroquinone based determination of electron and superoxide radical formed in heterogeneous photocatalytic systems. J. Photochem. Photobiol. A **407**, 113057. (10.1016/j.jphotochem.2020.113057

[50] Abdollahi Y et al. 2012 Photocatalytic degradation of 1,4-benzoquinone in aqueous ZnO dispersions. J. Braz. Chem. Soc. **23**, 236-240. (10.1590/S0103-50532012000200007)

[51] Sobczyński A, Duczmal Ł, Dobosz A. 1999 Photocatalysis by illuminated titania: oxidation of hydroquinone and p-benzoquinone. Monatshefte fuer Chemie **130**, 377-384. (10.1007/PL00010219)

[52] Su R et al. 2012 Promotion of phenol photodecomposition over TiO_2_ using Au, Pd, and Au–Pd Nanoparticles. ACS Nano **6**, 6284-6292. (10.1021/nn301718v) 22663086

[53] Szabó, V.; Mészáros R, Kónya Z, Kukovecz Á, Pálinkó I, Sipos P, Szabados M. 2022 Preparation and characterization of MnIn-layered double hydroxides (LDHs), extension of the synthesis to fabricate MnM(III)-LDHs (M = Al, Sc, Cr, Fe, Ga), and the comparison of their photocatalytic and catalytic activities in the oxidation of hydroquinone. J. Mol. Struct. **1261**, 132966. (10.1016/j.molstruc.2022.132966)

[54] Piszter G, Kertész K, Nagy G, Baji Z, Endre Horváth Z, Bálint Z, Sándor Pap J, Péter Biró L. 2022 Spectral tuning of biotemplated ZnO photonic nanoarchitectures for photocatalytic applications. Figshare. (10.6084/m9.figshare.c.6066566) PMC9277245 35845847

[55] Piszter G, Kertész K, Nagy G, Baji Z, Endre Horváth Z, Bálint Z, Sándor Pap J, Péter Biró L. 2022 Data from: spectral tuning of biotemplated ZnO photonic nanoarchitectures for photocatalytic applications. Dryad Digital Repository. (10.5061/dryad.w9ghx3fr8) PMC9277245 35845847

